# Comparison of parametric and non‐parametric Bayesian inference for fusing sensory estimates in physiological time‐series analysis

**DOI:** 10.1049/htl2.12003

**Published:** 2021-02-23

**Authors:** Tingting Zhu, Hamza Javed, David A. Clifton

**Affiliations:** ^1^ Institute of Biomedical Engineering Department of Engineering University of Oxford Oxford United Kingdom

## Abstract

The rapid proliferation of wearable devices for medical applications has necessitated the need for automated algorithms to provide labelling of physiological time‐series data to identify abnormal morphology. However, such algorithms are less reliable than gold‐standard human expert labels (where the latter are typically difficult and expensive to obtain), due to their large inter‐ and intra‐subject variabilities. Actions taken in response to these algorithms can therefore result in sub‐optimal patient care. In a typical scenario where only unevenly sampled continuous or numeric estimates are provided, without access to the “ground truth”, it is challenging to choose which algorithms to trust and which to ignore, or even how to merge the outputs from multiple algorithms to form a more precise final estimate for individual patients. In this work, the novel application of two previously proposed parametric fully‐Bayesian graphical models is demonstrated for fusing labels from (i) independent and (ii) potentially correlated algorithms, validated on two publicly available datasets for the task of respiratory rate (RR) estimation. These unsupervised models aggregate RR labels and estimate jointly the assumed bias and precision of each algorithm. Fusing estimates in this way may then be used to infer the underlying ground truth for individual patients. It is shown that modelling the latent correlations between algorithms improves the RR estimates, when compared to commonly employed strategies in the literature. Finally, it is demonstrated that the adoption of a strongly Bayesian approach to inference using Gibbs sampling results in improved estimation over the current state‐of‐the‐art (e.g. hierarchical Gaussian processes) in physiological time‐series modelling.

## 1 INTRODUCTION AND RELATED WORK

With the rapid increase in the volume and variety of wearable devices now routinely in use for healthcare applications, there exists the possibility of personalising the care patients receive based on their individual physiologies. This “personalised” and patient‐centric approach to healthcare is built on the assumption that physiological data collected from patient‐worn sensors can be reliably utilised, for diagnostic and prognostic applications, in clinical practice. However, with very large quantities of sensor data being accumulated over time, there is an urgent need for algorithms capable of automatically labelling the collected physiological time series data (e.g. abnormal respiratory rate readings) without the need for human input.

Yet to date, automated algorithms remain less reliable in practice than labelling from human experts. The latter is often the accepted gold‐standard but is typically expensive, difficult or even unfeasible to obtain for the majority of applications, such as labeling of data arising from patients in real‐time. In these cases, and many other real‐life clinical applications, automated algorithms have to be relied on to process and label sensor data. Additionally, when there is no knowledge of the “ground‐truth” in the form of expert labelling, it is a challenge to know which algorithms to trust and which to ignore at any given point in time. Particularly, as different algorithms may be optimal for different patient subsets, or even optimal for the same patient at different points in time. Often, naive methods are used to combine the recommendations of various algorithms to form a final estimate that is intended to have maximum precision for an individual.

Modelling continuous‐valued labels in addition to the biases and expertise of each annotator producing those labels, remains an active area of research, with key contributions outlined as follows. In the context of medical imaging [1], the use of an expectation maximisation (EM) method was demonstrated to fuse labels from different annotators estimating the diameter of lesions from images. A method for validating medical image segmentation, which estimated both the bias and variance of annotators, was proposed in the work of [2]. Similar to this approach, [3] more recently presented a model that estimated the ground truth in the form of count and percentage estimation, in a “crowd sensing” setting. A Bayesian EM framework which fused binary, multi‐valued and continuous‐valued labels was proposed in [4]. This method described explicitly modelling the precision (but not bias) of individual annotators by taking into account their different skill levels. By contrast, [5] used a Gaussian prior on the bias parameter of annotators attempting to produce cardiac landmark labels in 2D images. However, it is worth noting that physiological features were not incorporated into the models of [5] as a means of further improving estimation of the ground truth label.

In all of the aforementioned studies, the proposed models did not include a principled way to take into account the quality of data or how to cater for missing labels. Moreover, in these studies it was assumed that all annotators are independent, which may not always be the case when labels are produced by slightly different implementations of the same underlying algorithm. Previous work by the author tackled these issues by first proposing a Bayesian framework to jointly model both annotator bias and precision [6]. This work was then extended in [7], in which the author proposed a fully Bayesian approach through Gibbs sampling for fusing continuous valued labels, from both independent and/or partially correlated annotators, as a means of arriving at a consensus in an unsupervised manner.

In the case where we have imperfect algorithms that perform well for only some individuals for only some of the time, both parametric and non‐parametric models can be used to form a consensus. This guarantees an improvement of estimates over each algorithm considered independently. Bayesian models previously proposed in [7] are parametric, where the number of parameters is fixed and does not scale when the number of samples increases. They also explicitly model each timestamp in a time‐series and assume the data points in different timestamps are independent of each other. By contrast, a hierarchical Gaussian process [8], as a popular non‐parametric model for timeseries modelling, assumes the number of parameters scales with the dimension of the data, and explicitly models the correlation of datapoints among different timestamps in timeseries. Currently, there is no direct comparison of the aforementioned methods provided in the literature for physiological data, despite their similarity in model formulation.

In this letter, we present a novel application of the methodologies proposed by the author in [7]. We propose to compare the performance of both parametric and non‐parametric models and determine experimentally which method is more suitable for modelling physiological time‐series data in the case when combining multiple imperfect algorithms to form a consensus, using the two public datasets as exemplars. The task considered is estimation of the underlying respiratory rate (RR) from photoplethysmogram (PPG) recordings contained within the CapnoBase and BIDMC datasets [9, 10]. Robust estimation of RR is a practically well motivated task, as accurate monitoring of the vital sign can facilitate improved diagnosis and patient care. We demonstrate improved estimation of RR is possible using our approach of fusing labels from different annotators, when compared with existing methods presented in the literature; namely two EM models by [1] and [2], as well as a hierarchical Gaussian process approach [8].

The remainder of this letter is organised as follows. First, we outline the methodology proposed by the authors in [7], briefly describing the formulation of the two models considered. The experiments used to validate and compare the methods with selected baselines, along with the results obtained, are then detailed before the concluding remarks are presented.

## 2 PROBLEM FORMULATION

Consider the case where we have N samples of physiological time‐series data, with N corresponding continuous‐valued labels (e.g. RR labels from PPG time‐series samples). We can assume that the underlying ground truth for the $i^{\text{th}}$ sample, $z_{i}$, can be drawn from a Gaussian distribution with mean $a_{i}$ and variance 1/b. We can express $a_{i}$ as a linear regression function $f(w,x_{i})$ with an intercept $w_{0}$. In this formulation, w are the coefficients of the regression (which includes $w_{0}$ 1). While $x_{i}$ is a column feature vector for the $i^{\text{th}}$ record containing d features (i.e. we have an (N×d)‐dimensional design matrix, $X=[x_{1}^{⊺};\ldots ;x_{N}^{⊺}]$). Note that, a scalar value of one was added to the feature matrix (i.e. $x_{i}:=\left[1,x_{i}\right]$)) to cater for the $w_{0}$ intercept. Finally, the precision of the ground truth (defined as the inverse‐variance b) is assumed to be modelled from a gamma distribution where $k_{b}$ is the shape parameter and $\vartheta_{b}$ is the scale parameter. It therefore follows that the conditional probability density function (pdf) of z as a vector of labels can be written as $\prod_{i=1}^{N}N\left(z_{i}|x_{i}^{⊺}w,1/b\right)$.

## 3 THE INDEPENDENT ANNOTATOR MODEL (IAM)

Assuming once again the presence of N samples, we have a dataset, $D={\left[x_{i}^{⊺},y_{i}^{j=1},\ldots ,y_{i}^{j=R}\right]}_{i=1}^{N}$, where $y_{i}^{j}$ corresponds to the label estimate provided by the $j^{\text{th}}$ annotator for the $i^{\text{th}}$ sample, with a total of R annotators. This model assumes that $y_{i}^{j}$ is a noisy version of $z_{i}$, with a Gaussian distribution $N(y_{i}^{j}|z_{i},1/\lambda^{j})$, where $\lambda^{j}$ is the precision of the $j^{\text{th}}$ annotator, defined as the estimated inverse‐variance for annotator j. Furthermore, the bias of each annotator, which measures the average difference between the estimation and the ground truth, can be modelled as an additional term, denoted as $\phi^{j}$. The pdf of estimating $y_{i}^{j}$ can thus be written as $N(y_{i}^{j}|z_{i}+\phi^{j},1/\lambda^{j})$. It is assumed that $y_{i}^{1},\ldots ,y_{i}^{R}$ are conditionally independent given the ground truth $z_{i}$; assuming samples are independent, it follows that the conditional pdf of y can be expressed as:

(1) $$p\left(y|z,\phi ,\lambda \right)=\prod_{i=1}^{N}\prod_{j=1}^{R}N\left(y_{i}^{j}|z_{i}+\phi^{j},1/\lambda^{j}\right).$$

However, as noted earlier, conditional independence between annotators may not always be the case as labels may be produced by variants of the same underlying algorithmic approach. That is annotators that differ only in, for example, operational parameter settings. Nevertheless, this assumption can be made to simplify the model and subsequent derivation of the likelihood. Relaxation of this independence assumption will be explored in the second proposed model, the correlated annotator model (described in the proceeding section). The pdf of the bias for annotator j, $\phi^{j}$, is assumed to once again be drawn from a Gaussian, this time with mean $\mu_{\phi }$ and variance $1/\alpha_{\phi }$ [5]:

(2) $$p\left(\phi^{j}|\mu_{\phi },\alpha_{\phi }\right)=N\left(\phi^{j}|\mu_{\phi },1/\alpha_{\phi }\right).$$

Although the biases of the annotators could very well be assumed to follow other distributions, such choices are likely to be dataset‐dependent. In the absence of any knowledge of the underlying distribution of biases, we choose to assume they are drawn from a Gaussian distribution. The precision values (defined as the inverse‐variance values, constrained to be in the range of (0,∞)), such as $\lambda^{j}$ and $\alpha_{\phi }$, by contrast are assumed to be drawn from a gamma distribution, with parameters $k_{\lambda }$, $\vartheta_{\lambda }$, and $k_{\alpha }$, $\vartheta_{\alpha }$, respectively:

(3) $$p\left(\lambda^{j}|k_{\lambda },\vartheta_{\lambda }\right)=\mathrm{Gamma}\;\left(\lambda^{j}|k_{\lambda },\vartheta_{\lambda }\right),$$

(4) $$p\left(\alpha_{\phi }|k_{\alpha },\vartheta_{\alpha }\right)=\mathrm{Gamma}\;\left(\alpha_{\phi }|k_{\alpha },\vartheta_{\alpha }\right).$$

It follows that for a given dataset D, the likelihood of the parameters $\theta =\{w,\lambda ,\phi ,\alpha_{\phi },b,z_{i}\}$, can be formulated as:

(5) $$p\left(D|\theta \right)=\prod_{i=1}^{N}p\left(y_{i}^{1},\ldots ,y_{i}^{R},x_{i}|\theta \right).$$

Bayes' theorem can then be used to determine the posterior probability of the parameters θ, for a given dataset D, as

(6) $$p\left(\theta |D\right)=\frac{p\left(D|\theta \right)p\left(\theta \right)}{\int_{\theta }p\left(D|\theta \right)p\left(\theta \right)d\theta },$$

where

$$\begin{array}{cc} & p\left(D|\theta \right)p\left(\theta \right)\\ & =\mathrm{Gamma}\left(\alpha_{\phi }|k_{\alpha },\vartheta_{\alpha }\right)\mathrm{Gamma}\left(b|k_{b},\vartheta_{b}\right)\\ & \;\times \;\left[\prod_{j=1}^{R}N\left(\phi^{j}|\mu_{\phi },1/\alpha_{\phi }\right)\mathrm{Gamma}\left(\lambda^{j}|k_{\lambda },\vartheta_{\lambda }\right)\right]\\ & \;\times \;\left[\prod_{i=1}^{N}N\left(z_{i}|x_{i}^{{}^{⊺}}w,1/b\right)\prod_{j=1}^{R}N\left(y_{i}^{j}|z_{i}+\phi^{j},1/\lambda^{j}\right)\right]. \end{array}$$

Obtaining the posterior probability of the parameters θ effectively allows us to estimate the latent ground truth for the $i^{\text{th}}$ sample $z_{i}$, and jointly predict the bias $\phi^{j}$ and precision $\lambda^{j}$ of the $j^{\text{th}}$ annotator simultaneously.

## 4 LEARNING FROM INCOMPLETE DATA USING GIBBS SAMPLING

**FIGURE 1:**
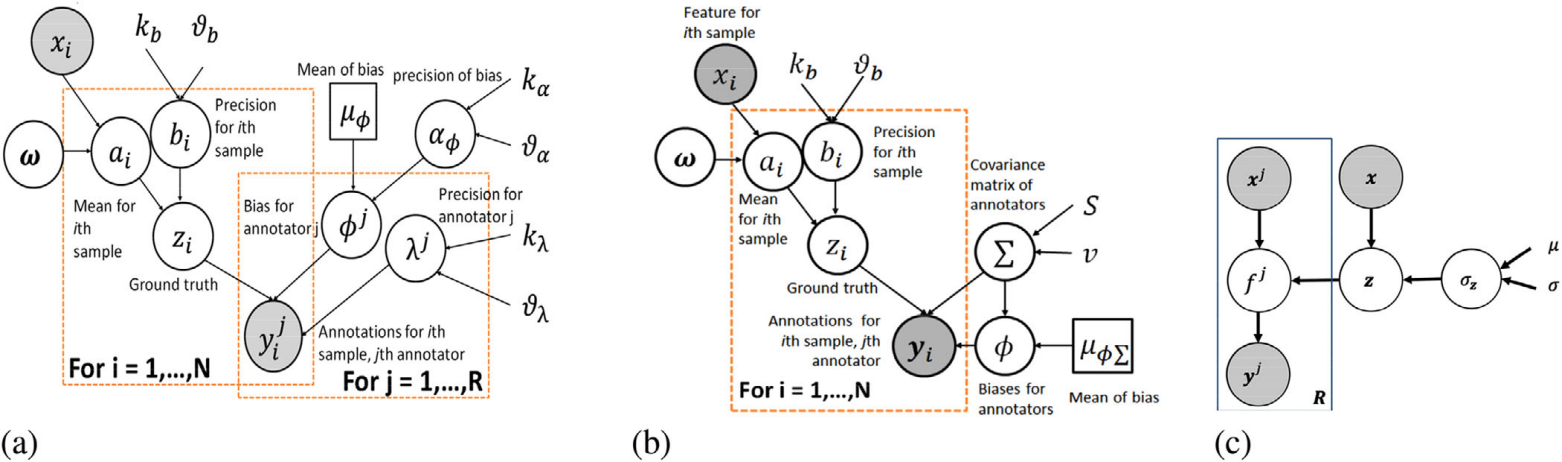
(a) The independent annotator model. (b) The correlated annotator model. (c) Hierarchical Gaussian processes with an additional prior on the latent ground truth

An important practical scenario to consider is the case that arises when there are missing labels from different annotators (i.e. not all R algorithms provide N estimates for all samples). To account for this, the posterior distribution hyperparameters of the IAM can be re‐written using Gibbs sampling as follows (see graphical model in Figure 1(a)):

$$\begin{array}{cc} z_{i} & ∼N\left(z_{i}|a_{i}^{*},\frac{1}{b_{i}^{*}}\right),\phi^{j}∼N\left(\phi^{j}|\mu_{\phi }^{j*},\frac{1}{\alpha_{\phi }^{j*}}\right),\\ \lambda^{j} & ∼\mathrm{Gamma}\left(\lambda^{j}|k_{\lambda }^{j*},\vartheta_{\lambda }^{j*}\right),\\ b & ∼\mathrm{Gamma}\left(b|k_{b}^{*},\vartheta_{b}^{*}\right),\alpha_{\phi }∼\mathrm{Gamma}\left(\alpha_{\phi }|k_{\alpha }^{*},\vartheta_{\alpha }^{*}\right). \end{array}$$

$$\begin{array}{cc} & a_{i}^{*}=\frac{\left(x_{i}^{⊺}w\right)b+\sum_{j\in V_{i}}\left[\left(y_{i}^{j}-\phi^{j}\right)\lambda^{j}\right]}{b+\sum_{j\in V_{i}}\lambda^{j}},b_{i}^{*}=b+\sum_{j\in V_{i}}\lambda^{j}, \end{array}$$

$$\begin{array}{cc} & \mu_{\phi }^{j*}=\frac{\mu_{\phi }\alpha_{\phi }+\lambda^{j}\sum_{i\in U_{j}}\left(y_{i}^{j}-z_{i}\right)}{\alpha_{\phi }+\sum_{i\in U_{j}}\lambda^{j}},\alpha_{\phi }^{j*}=\alpha_{\phi }+\sum_{i\in U_{j}}\lambda^{j},\\ & k_{\lambda }^{j*}=k_{\lambda }+\frac{N_{j}}{2},\frac{1}{\vartheta_{\lambda }^{j*}}=\frac{\sum_{i\in U_{j}}{\left(y_{i}^{j}-\phi^{j}-z_{i}\right)}^{2}}{2}+\frac{1}{\vartheta_{\lambda }},\\ & k_{\alpha }^{*}=k_{\alpha }+\frac{R}{2},\frac{1}{\vartheta_{\alpha }^{*}}=\frac{\sum_{j=1}^{R}{\left(\phi^{j}-\bar{\phi }\right)}^{2}}{2}+\frac{1}{\vartheta_{\alpha }},\\ & k_{b}^{*}=k_{b}+\frac{N}{2},\frac{1}{\vartheta_{b}^{*}}=\frac{\sum_{i=1}^{N}{\left(z_{i}-\bar{z}\right)}^{2}}{2}+\frac{1}{\vartheta_{b}}. \end{array}$$

Note that $U_{j}$ is the set of samples with labels provided by the $j^{\text{th}}$ annotator whilst $V_{i}$ is the set of annotators that provided labels for the $i^{\text{th}}$ sample, and $N_{j}$ is the number of samples annotated by the $j^{\text{th}}$ annotator. Finally, w can be learnt by finding the zero gradient of the expectation of the complete data log‐likelihood as $w={\left(\sum_{i=1}^{N}x_{i}x_{i}^{⊺}\right)}^{-1}\sum_{i=1}^{N}x_{i}z_{i}$. The above formulation allows us to cope robustly with the commonly encountered difficulties arising from incomplete (or even sparse) labelling, in a principled and probabilistic manner.

## 5 THE CORRELATED ANNOTATOR MODEL (CAM)

As noted previously, annotator independence may not always be an accurate assumption to make in reality. To account for this, we can incorporate a correlation measure into the annotator model described in the preceding section. This would facilitate an improved aggregation of the different annotator labels, and thus a better inferred ground truth estimate. In this formulation, annotators are considered to be anomalous when they are highly correlated to other annotators but possess relatively large variances and biases. These anomalous annotators are penalised with lower weighting for their labels. Expert annotators, defined as those that are highly correlated to other annotators but which have relatively small variances and biases, on the other hand have their labels weighted more heavily in the model.

A multivariate normal distribution (MVN) can be applied to the annotator model, using the covariance matrix (denoted Σ) to describe the correlation among annotators, as well as providing a constraint on the biases ϕ. The Inverse‐Wishart (IW) distribution is used as a prior for the covariance matrix Σ, and the bias values ϕ for all annotators are modelled using a MVN with mean $\mu_{\phi \Sigma }$ and covariance $\Sigma /k_{0}$. The conditional pdf of the modified annotator model with covariance becomes

(7) $$p\left(y|z_{i},\phi ,\Sigma \right)=\prod_{i=1}^{N}N\left(z_{i}+\phi ,\Sigma \right),$$

where Σ is the covariance matrix of the R annotators and where there are N samples.

Matrix Σ can be further decomposed into a correlation matrix and the precision values of the annotators. Using the separation strategy proposed by [11], Σ is formulated as Σ=QρQ, where Q is an R‐by‐R diagonal matrix with entries being $\frac{1}{\sqrt{\lambda^{j=1}}},\ldots ,\frac{1}{\sqrt{\lambda^{j=R}}}$. Here, $\lambda^{j}$ is the precision value for the $j^{\text{th}}$ annotator, and ρ is the latent correlation matrix of the annotation errors among R annotators. The biases of individual annotators are now assumed to be drawn from a MVN constrained by Σ, with conditional pdf:

(8) $$p\left(\phi |\mu_{\phi \Sigma },\Sigma \right)=N\left(\phi |\mu_{\phi \Sigma },\Sigma /k_{0}\right),$$

where $\mu_{\phi \Sigma }$ is the prior mean for ϕ, and $k_{0}$ is a positive scalar that expresses our belief on $\mu_{\phi \Sigma }$. The posterior of the parameter $\theta_{c}=\left\{\phi ,\Sigma ,b,z_{i}\right\}$ for a given dataset D can be written using Bayes' theorem as

(9) $$\begin{array}{c} p\left(\theta_{c}|D\right)=\frac{p\left(D|\theta_{c}\right)p\left(\theta_{c}\right)}{\int_{\theta_{c}}p\left(D|\theta_{c}\right)p\left(\theta \right)d\theta_{c}}, \end{array}$$

where:

$$\begin{array}{cc} & p\left(D|\theta_{c}\right)p\left(\theta_{c}\right)\\ & =N\left(\phi |\mu_{\phi \Sigma },\Sigma /k_{0}\right)\mathrm{IW}\left(\Sigma |v,S\right)\\ & \times \mathrm{Gamma}\;\left(b|k_{b},\vartheta_{b}\right)\left[\prod_{i=1}^{N}N\left(z_{i}|a_{i},1/b\right)N\left(y_{i}|z_{i}+\phi ,\Sigma \right)\right]. \end{array}$$

The new parameters are now updated using the Gibbs sampler as follows (see graphical model in Figure 1(b)) :

$$\begin{array}{cc} & \phi ∼N\left(\phi |\mu_{\phi \Sigma }^{*},\Sigma_{\phi }^{*}\right),\Sigma ∼\mathrm{IW}\left(\Sigma |v^{*},S^{*}\right). \end{array}$$

$$\begin{array}{cc} & \mu_{\phi \Sigma }^{*}=\frac{k_{0}\mu_{\phi \Sigma }}{k_{0}+N}+\frac{U{\bar{y}}_{b}}{k_{0}+U},\Sigma_{\phi }^{*}=\frac{\Sigma }{k_{0}+N},v^{*}=v+N,\\ S^{*} & =S+\sum_{i=1}^{N}{\left(y_{i}-z_{i}-{\bar{y}}_{b}\right)}^{T}\left(y_{i}-z_{i}-{\bar{y}}_{b}\right)\\ & +\frac{k_{0}N}{k_{0}+N}{\left({\bar{y}}_{b}-\mu_{\phi \Sigma }\right)}^{T}\left({\bar{y}}_{b}-\mu_{\phi \Sigma }\right), \end{array}$$

where U is a 1‐by‐R vector, and each of its elements indicates the total number of labels provided by a respective annotator. ${\bar{y}}_{b}=\left[{\bar{y}}_{b}^{j=1},\ldots ,{\bar{y}}_{b}^{j=R}\right]$, where ${\bar{y}}_{b}^{j}=\frac{1}{N_{j}}\sum_{i=1}^{N}\left(y_{i}^{j}-z_{i}\right)$.

## 6 EXPERIMENT DESCRIPTION

We evaluate the efficacy of our proposed models using two publicly available biomedical datasets: (1) the CapnoBase dataset by [9] and (2) the BIDMC dataset by [10]. The CapnoBase dataset contains 42 PPG recordings of spontaneous or controlled breathing from a total of 42 subjects (29 paediatric and 13 adults), where each recording is 8 min long. The BIDMC dataset contains PPG recordings of the same duration, from 53 adult subjects. A subject's RR manifests itself in the PPG recordings by modulating the PPG waveform signal. For our experiments, we extract three respiratory‐induced modulation time‐series (Amplitude Modulation, Baseline Wander, and Frequency Modulation) from each of the PPG recordings. Then to estimate RR from these time‐series, we used two well established approaches: Fourier spectral analysis and autoregressive (AR) modelling. The RR estimates were computed for 32‐s windows, with successive windows having 29 s overlap. For each window and each method (AR and Fourier spectral analysis), three RR estimates were calculated from the three modulation time‐series, producing six RR estimates for every window. The underlying subject‐specific latent RR was then estimated by fusing these six estimation “algorithms”.

## 7 RESULTS AND DISCUSSION

We compared our proposed models with two parametric maximum‐likelihood EM models (EM‐R by [1]; STAPLE by [2]), as well as the non‐parametric hierarchical Gaussian processes (HGPs) ([8]) with an additional Bayesian regularisation (i.e. a lognormal prior) on the noise variance of the latent ground truth (see Figure 1(c)). By comparing the gold‐stand RR labels for a subject over 150 windows, the mean absolute error (MAE) was computed for each model. The mean MAE and the standard error of the mean (SEM) were also estimated across all subjects. The results are shown in Table 1. The CAM had the least error for CapnoBase, but the IAM model was better for BIDMC. Nevertheless, both proposed models outperformed the state‐of‐the‐art approaches recreated from the literature: a MAE of 1 bpm versus 1.5 bpm [10] and 1.2 bpm [9] for the CapnoBase dataset, and a MAE of 2.96 bpm versus 4 bpm [10] and 5.8 bpm [9] for the BIDMC datase using all possible windows.

**TABLE 1:** Mean MAE ± SEM (bpm) of the inferred RR across subjects using different models for CapnoBase and BIDMC datasets

Model	CapnoBase	BIDMC
EM‐R	1.14 ± 0.22	3.15 ± 0.34
sSTAPLE	1.78 ± 0.34	3.51 ± 0.28
HGPs	1.46 ± 0.32	3.37 ± 0.40
IAM	1.18 ± 0.16	2.96 ± 0.43
CAM	1.00 ± 0.20	3.03 ± 0.33

**FIGURE 2:**
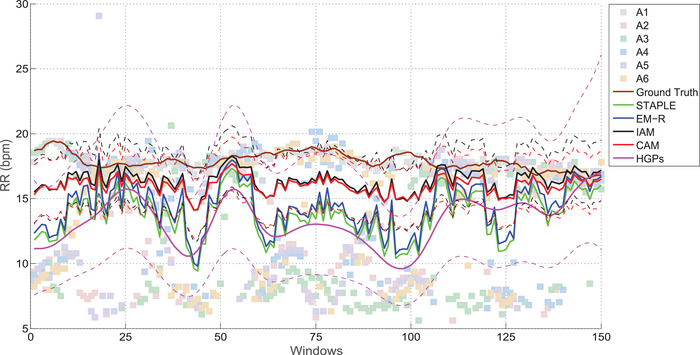
Example of the RR estimates for a subject

Furthermore, the proposed parametric models provide the Bayesian interpretation of their estimates through 95% confidence intervals (see dashed lines in Figure 2). In comparison, HGPs had a larger noise variance: as 5 out of 6 algorithms (A2 to A6) were biased with smaller estimation of RR, this resulted in a large uncertainty in the latent RR estimates when fusing labels using HGPs. Although HGPs models the time‐dependent information of the RR time‐series, it operates under the assumption that estimates from the algorithms are reliable and equally weighted. This is not the case when algorithms are biased and affected by noise and artefacts, and hence causing HGPs to have sub‐optimal performance when compared to IAM or CAM. In terms of model complexity, both HGPs and the proposed parametric models are in the order of $O(n^{3})$, where n is the number of samples. Additional reduction in computational complexity would be possible through further optimising the inference steps, which can be explored in future work.

## 8 CONCLUSION

Automated labelling of large volumes of physiological time‐series data being collected from wearable sensors, often in real‐time, is a prerequisite to being able to provide patients with personalised care. In this work, we have applied two parametric unsupervised fully Bayesian graphical models for fusing labels from (i) independent and (ii) potentially correlated algorithms, to estimate the underlying RR from PPG signals obtained from the publicly available CapnoBase and BIDMC datasets. Robust estimation of RR is of clinical value and could be used to improve patient care. By jointly estimating the assumed bias and precision of each algorithm considered, we have demonstrated that these models are able to infer the underlying ground truth more robustly than existing state‐of‐the‐art methods. In addition to improved performance, we show that the proposed models are robust when dealing with missing values (as often occurs in real‐life biomedical applications due to sensor failure), and that they are suitably efficient for use in real‐time applications.

## Footnotes

1
$w_{0}$ models the overall offset predicted in the regression, and is therefore different from the bias ϕ specific to each annotator in the proposed models, which will be described later.
